# Characterization, Genomic Organization, Abundance, and Chromosomal Distribution of Ty1-copia Retrotransposons in *Erianthus arundinaceus*

**DOI:** 10.3389/fpls.2017.00924

**Published:** 2017-06-07

**Authors:** Yongji Huang, Ling Luo, Xuguang Hu, Fan Yu, Yongqing Yang, Zuhu Deng, Jiayun Wu, Rukai Chen, Muqing Zhang

**Affiliations:** ^1^National Engineering Research Center for Sugarcane, Fujian Agriculture and Forestry UniversityFuzhou, China; ^2^Guangxi Collaborative Innovation Center of Sugar Industries, Guangxi UniversityNanning, China; ^3^Guangdong Key Laboratory of Sugarcane Improvement and BiorefineryGuangzhou, China; ^4^Guangdong Provincial Bioengineering Institute, Guangzhou Sugarcane Industry Research InstituteGuangzhou, China

**Keywords:** *Erianthus arundinaceus*, Ty1-copia retrotransposons, phylogenetic diversity, genomic abundance, chromosomal distribution

## Abstract

*Erianthus arundinaceus* is an important wild species of the genus *Saccharum* with many valuable traits. However, the composition and structure of its genome are largely unknown, which have hindered its utilization in sugarcane breeding and evolutionary research. Retrotransposons constitute an appreciable fraction of plant genomes and may have played a significant role in the evolution and sequence organization of genomes. In the current study, we investigate the phylogenetic diversity and genomic abundance of Ty1-copia retrotransposons for the first time and inspect their chromosomal distribution patterns in *E. arundinaceus*. In total, 70 Ty1-copia reverse transcriptase (RT) sequences with significant levels of heterogeneity were obtained. The phylogenetic analysis revealed these Ty1-copia retrotransposons were classified into four distinct evolutionary lineages (Tork/TAR, Tork/Angela, Retrofit/Ale, and Sire/Maximus). Dot-blot analysis showed estimated the total copy number of Ty1-copia retrotransposons to be about 4.5 × 10^3^ in the *E. arundinaceus* genome, indicating they were a significant component. Fluorescence *in situ* hybridization revealed that Ty1-copia retrotransposons from the four lineages had strikingly similar patterns of chromosomal enrichment, being exclusively enriched in the subterminal heterochromatic regions of most *E. arundinaceus* chromosomes. This is the first clear evidence of the presence of Ty1-copia retrotransposons in the subterminal heterochromatin of *E. arundinaceus*. Altogether, these results promote the understanding of the diversification of Ty1-copia retrotransposons and shed light on their chromosomal distribution patterns in *E. arundinaceus*.

## Introduction

*Erianthus* *arundinaceus* is a perennial bunch grass with many valuable traits, such as strong rooting and tillering ability, wide adaptability and doughty resistance to biotic and abiotic stress (Jackson and Henry, 2011). Therefore, it has been recognized as one of the most important wild species to the genus *Saccharum* for increasing the narrow genetic basis of modern sugarcane cultivars (Jackson and Henry, 2011). The genetic diversity of *E. arundinaceus* has been successfully introgressed into sugarcane backgrounds, and a series of genuine progeny have been produced in different backcrossed generations (D’Hont et al., 1995; Piperidis et al., 2001, 2010; Cai et al., 2005; Wu et al., 2014; Huang et al., 2015). The genomic size of *E. arundinaceus* had been estimated to be about 3.57 Gb per haploid chromosome equivalent (Yan et al., 2016). However, due to the lack of genomic sequence data, the evolution and sequence organization of the *E. arundinaceus* genome remain largely unknown.

In eukaryotes, transposable elements are the predominant class of movable genetic elements that can alter their chromosomal locations in host genomes. They represent a major fraction of interspersed repetitive DNA in eukaryotes, especially those of higher plant species. According to their transposition mechanisms, transposable elements are split into two classes, DNA transposons and retrotransposons (Wicker et al., 2007). Retrotransposons are the most abundant transposable elements in plants and play important roles in genome evolution because of their large size and potential for massive increases in copy number. Retrotransposons transpose via an RNA intermediate through a copy-and-paste mechanism. Based on whether they have long terminal repeat (LTR), retrotransposons can be further classified into two distinct types, LTR retrotransposons and non-LTR retrotransposons. LTR retrotransposons are structurally and phylogenetically close to retroviruses, flanking the genes encoding a core protein and a polyprotein. The polyprotein consists of four domains: protease, integrase, reverse transcriptase (RT), and ribonuclease H (Wilhelm and Wilhelm, 2001). LTR retrotransposons are divided into five subclasses: Ty1-copia, Ty3-gypsy, Bel-Pao, retroviruses, and endogenous retroviruses (Kumar and Bennetzen, 1999; Kumar and Bennetzen, 2000). Of these, the large Ty1-copia and Ty3-gypsy subclasses are present in almost all eukaryotic genomes and have already been extensively studied, whereas members of the last three subclasses have been detected only in metazoan hosts (Wicker et al., 2007; Chaux and Wagner, 2011). The main difference between Ty1-copia and Ty3-gypsy is the order of encoded components within the polyprotein domain. In Ty1-copia types, these subunits are arranged in the order protease, integrase, RT, and ribonuclease H, while the order is protease, RT, ribonuclease H, and integrase in Ty3-gypsy types.

In the past decade, the increasing availability of genome sequences is providing unprecedented opportunities for understanding the patterns of variation existing among the entire complement of retrotransposons in the plant kingdom (Paterson et al., 2009; Schnable et al., 2009; Chen et al., 2013; Kawahara et al., 2013; Choulet et al., 2014; Mayer et al., 2014). It has been suggested that genome expansion is largely attributed to retrotransposon amplification. In *Oryza brachyantha*, a wild relative of rice (*O. sativa*), only ∼10% of the nuclear genome is composed of retrotransposons. Low activity for LTR-retrotransposon proliferation and massive removal of ancient LTR retrotransposons lead to such a small genome in *O. brachyantha* (261 Mb) (Chen et al., 2013). In contrast to *O. brachyantha*, due to two recent bursts of LTR retrotransposons, LTR retrotransposons make up over 70% of the nuclear genome in *O. sativa* (Kawahara et al., 2013). In the past several million years, maize has undergone extensive and periodic amplifications of retrotransposons, ∼79% of the nuclear genome with a moderately large genome (∼2400 Mb) is composed of LTR retrotransposons (Schnable et al., 2009). In sorghum, ∼55% of the nuclear genome (750 Mb) is composed of retrotransposons, higher than the genome of *O. sativa*, but fewer than the maize genome (Paterson et al., 2009). Regardless of the abundance of LTR retrotransposons in their host genomes, LTR retrotransposons have a tendency to be accumulated in the pericentromeric regions of many plant genomes, such as sorghum. In general, pericentromeric regions show suppressed genetic recombination and are gene-poor regions, in which preferential insertions of LTR retrotransposons would cause less frequent deleterious mutations than those inserted in gene-rich chromosomal arms. Nevertheless, a recent study revealed that, unlike in many other plant genomes, the young LTR retrotransposons in *Solanum lycopersicum* are preferentially distributed in euchromatic regions (Xu and Du, 2014).

In numerous plant genomes, the RT domains of the Ty1-copia retrotransposon have been frequently identified and investigated (Flavell et al., 1992; Pearce et al., 1996b; Ma et al., 2008; Alipour et al., 2013; Kolano et al., 2013; Lee et al., 2013). Since Ty1-copia RT domains contain several highly conserved amino acid sequences, degenerate primers designed from highly conserved sequences have been used to amplify these domains by polymerase chain reaction (PCR). The advent of PCR methods for amplifying Ty1-copia RT domains has led to the rapid increase of finding on sequence evolution and phylogenetic relationship, both within and among different plant taxa (Flavell et al., 1992; Pearce et al., 1996b; Goodwin and Poulter, 2002; Santini et al., 2002; Ma et al., 2008; Alipour et al., 2013; Kolano et al., 2013; Lee et al., 2013). In addition, dot-blot hybridization has been used to quantitatively assess the relative abundance of Ty1-copia RT sequences in host genomes (Ma et al., 2008; Kolano et al., 2013). Moreover, the chromosomal localization of Ty1-copia RT sequences is also an important criterion when studying the retrotransposition dynamics of their retrotransposons. Fluorescence *in situ* hybridization (FISH) is an ideal technique to investigate the chromosomal distribution of Ty1-copia RT sequences in plant genomes (Pearce et al., 1996b; Domingues et al., 2012; Alipour et al., 2013; Kolano et al., 2013; Lee et al., 2013). Detailed characterizations of the content, variability, copy number, and chromosomal distribution patterns of Ty1-copia RT sequences in different host species have provided some insight into their genomic organization and evolution. However, lack of basic genome structure and evolutionary information has hindered utilization of these sequences in *E. arundinaceus* breeding and evolutionary research.

In the current study, we isolated, cloned, and sequenced Ty1-copia RT sequences from the *E. arundinaceus* genome with the goal of identifying retrotransposon sequences and examining their heterogeneity, phylogenetic relationship, genomic abundance, and chromosomal distribution patterns.

## Materials and Methods

### Plant Materials

*Erianthus arundinaceus* Hainan 92-77 (2*n* = 6*x* = 60) plants were grown in the greenhouse at Fujian Agricultural and Forestry University (Fuzhou, China). Total genomic DNA was extracted from young leaves based on a standard cetyltrimethyl ammonium bromide (CTAB) protocol (Doyle, 1987).

### PCR Amplification and Cloning of PCR Products

A pair of degenerate primers (forward: 5′-ACNGCNTTYYTNCAYGG-3′; reverse: 5′-ARCATRTCRTCNACRTA-3′) corresponding to highly conserved peptide sequence of the Ty1-copia RT domain were used to amplify RT domains of Ty1-copia retrotransposons (Flavell et al., 1992). PCR amplification was carried out on a Veriti 96-well Thermal Cycler (Applied Biosystems, Foster City, CA, United States). Each PCR reaction contained 50 ng of total genomic DNA, 20 pmol of each primer, 0.2 mM of each dNTP, 1 × ExTaq buffer, and 2.5 U of Ex Taq polymerase (Takara, Bio Inc., Tokyo, Japan) in a total volume of 50 μL. PCR reactions were performed using the following conditions: initial denaturation at 94°C for 3 min, 35 cycles of 94°C denaturation for 30 s, annealing at 50°C for 30 s, extension at 72 °C for 1 min, and final extension at 72°C for 10 min. To isolate the heterogeneous Ty1-copia RT sequences, three independent rounds of PCR amplification and cloning were conducted for Ty1-copia RT sequences. After purification by gel extraction, PCR amplification products were cloned into a pMD19-T vector (Takara, Japan) and then transformed into the DH5α strain of *Escherichia coli*. Positive recombinant clones were further confirmed by PCR with M13 primers. Randomly chosen recombinant colonies were selected to extract plasmid DNA. The resulting amplicons were sequenced bidirectionally (forward and reverse) using M13 universal primers from Beijing Genomics Institute Co, Ltd. (Shenzhen, China). In total, 70 Ty1-copia RT sequences were deposited in the GenBank database under accession numbers KY593344-KY593413, and these sequences were designated as EaTy1-copia-1 to EaTy1-copia-70, respectively.

### Phylogenetic Analysis

Ty1-copia RT nucleotide sequences from *E. arundinaceus* identified were compared with previously identified those from other graminaceous species (*Saccharum*, *Triticum*, *Hordeum*, *Oryza*, *Sorghum*, and *Zea*) in the NCBI GenBank database^1^ using blastn. Sequence similarity searches were conducted using blastn algorithm parameters, including automatically adjusting for short input sequences, as expected threshold of 10, word size of 28, match/mismatch scores of 1–2, linear gap costs, and regional low complexity filtering. To detect any sequence similarity with previously known elements from graminaceous species, the search algorithm had to be changed from megablast (highly similar sequences) to blastn (somewhat similar sequences). Nucleotide sequences were aligned using CLUSTALW as implemented by MEGA 7 (Kumar et al., 2016). All nucleotide sequences of Ty1-copia retrotransposons from *E. arundinaceus* were translated to amino acid sequences by the Transeq tool of the EMBOSS package^2^ (ExPASy Proteomics Tools). Ty1-copia RT amino acid sequences were aligned against other similar sequences from graminaceous species by using MACSE with default parameters (Ranwez et al., 2011), which were back translated to produce alignments of the original nucleotide sequences. MACSE can detect interruptions in open reading frames (ORFs) due to: (1) nucleotide substitutions that result in stop codons and (2) insertion or deletion of nucleotides (non-multiples of three) that induce frameshifts (Ranwez et al., 2011). To retain ORFs, gaps were introduced in the multiple sequence alignment and PCR primer regions were excluded from phylogenetic analysis. Multiple sequence alignment of the Ty1-copia RT sequences from *E. arundinaceus* and other graminaceous species was undertaken by using MUSCLE (Edgar, 2004). Phylogenetic analysis of the aligned Ty1-copia RT sequences based on p-distance and supported with 1000 bootstrap replicates, was performed using Neighbor-Joining method in MEGA 7 (Kumar et al., 2016). The pairwise deletion option for missing data and gaps was used throughout the distance analysis. Since the Department of Energy, Joint Genome Institute^3^ (Phytozome v12.0) has the genome sequence data from rice (*O. sativa* v7), sorghum (*Sorghum bicolor* v3.1), and maize (*Zea mays* PH207 v1.1), the data provide insights into the distribution and copy number of the Ty1-copia RT sequences in the three species. Blast searches with default parameters (Target type = Genome, Program = BLASTN – nucleotide query to nucleotide db, Expect threshold = -1, Comparison matrix = BLOSUM62, Word length = 11 for BLASTN and 3 for all others, # Of alignments to show = 100, Allow gaps, and Filter query) were conducted with Ty1-copia RT sequences in *E. arundinaceus* against those in the three species.

### Dot-Blot Hybridization

The relative abundance of isolated Ty1-copia RT sequences in Ty1-copia retrotransposons from the *E. arundinaceus* genome was determined using dot-blot hybridization relative to a positive control (*E. arundinaceus* genome DNA) and a negative control (double-distilled H_2_O). All purified plasmids containing clones of Ty1-copia RT domains were quantified in NanoVue Plus^TM^ (GE Healthcare, Princeton, NJ, United States) and then diluted to a final concentration of 20 ng/μL. These plasmids were denatured by heating to 100°C for 10 min, then quickly chilled in an ice/water bath. The denatured plasmids were transferred onto the Amersham Hybond-N^+^ nylon membrane (GE Healthcare, Life Sciences, Indianapolis, IN, United States). One microliter of each plasmid was spotted onto the membranes, and DNA was fixed to the membrane by UV crosslink using Stratalinker^TM^ UV Crosslinker (Stratagene, La Jolla, CA, United States). After fixation, the membrane was washed with sterile distilled water and air-dried. The digoxigenin-11-dUTP (DIG)-labeled probe was constructed from genomic DNA of *E. arundinaceus* Hainan 92-77 using a DIG Nick Translation Kit (Roche Diagnostics). Hybridization was performed overnight at 42°C following the manufacturer’s recommended protocol for the DIG High Prime DNA Labeling and Detection Starter Kit I (Roche Diagnostics). High stringency washes were performed following a rinse in wash solution containing 0.2 × saline-sodium citrate (SSC) and 0.1 % sodium dodecyl sulfate; then the blots were washed twice at 68°C for 15 min each. Hybridization signals were subsequently detected and quantified with ChemiDocXRS (Bio-Rad, Hercules, CA, United States). The Ty1-copia retrotransposon sequence copy number in *E. arundinaceus* was estimated by quantitative dot-blot hybridization of serial dilutions of genomic DNA and the complete heterogeneous population of sequences. The denatured genomic DNA and this population of sequences were serially diluted (500, 400, 300, 200, 100, and 50 ng), denatured for 10 min at 98°C, and then transferred to an Amersham Hybond-N^+^ nylon membrane (GE Healthcare). Ty1-copia sequences labeled with DIG were used as probes for the PCR-DIG Probe Synthesis Kit (Roche Diagnostics) following the manufacturer’s instructions. Hybridization was performed as described above. The copy number per genome was estimated by determining the hybridization intensity using ImageJ software (Schneider et al., 2012).

### FISH

The root tip meristems of *E. arundinaceus* Hainan 92-77 were pretreated with saturated *p*-dichlorobenzene solution at 25°C for 2 h to accumulate metaphase cells, fixed in 3:1 (v/v) ethanol:acetic acid for 24 h, and then stored at -20°C in 75% ethanol solution until use. The fixed roots were washed in water and digested in an enzyme solution containing 4% Onozuka R10 cellulose (Yakult, Tokyo, Japan), 0.5% pectolyase Y-23 (Yakult, Tokyo, Japan), and 0.5% pectinase (Sigma, St. Louis, MO, United States) at 37°C for 120 min. The meristematic cells of root tips were squashed on a clean slide in a drop of 3:1 (v/v) ethanol:acetic acid, then air-dried and stored at -20°C until use. FISH was performed as described by D’Hont et al. (1995) with minor modifications. Clones containing the isolated RT domain of *E. arundinaceus* were labeled with DIG by PCR with degenerate primers according to the PCR-DIG Probe Synthesis Kit (Roche Diagnostics) instructions. As a control, clones containing isolated non-Ty1-copia-related retrotransposons were labeled with biotin using PCR with degenerate primers. The hybridization mixture (total volume, 50 μL) containing 100 ng of the labeled DNA probe, 50% deionized formamide, 10% dextran sulfate and 2 × SSC was denatured at 97°C for 10 min. Slides were denatured in denaturation solution (70% deionized formamide and 2 × SSC) at 80°C for 3 min, dehydrated in a series of ice-cold ethanol solutions (70, 95, and 100% ethanol), and incubated overnight in a humid chamber at 37°C. Posthybridization washes were performed sequentially, once in 2 × SSC at 42°C for 5 min, twice in 20% deionized formamide and 2 × SSC at 42°C for 5 min, twice in 2 × SSC at 42°C for 5 min, once in 2 × SSC at 42 °C for 5 min, and once in 4 × SSC and 0.2% Tween-20 for 5 min at room temperature. Stringent washes were performed thrice in 4 × SSC and 0.2% Tween-20 at 37°C for 8 min. The DIG-labeled probes were detected by anti-DIG-FITC primary antibody (Roche Applied Science) and an FITC-conjugated anti-sheep secondary antibody (Jackson ImmunoResearch, Suffolk, United Kingdom), respectively. The biotin-labeled probe was detected with avidin D, rhodamine 600 (XRITC), and a biotinylated anti-avidin antibody (Vector Laboratories, Burlingame, CA, United States), respectively. After detection, chromosomes were counter-stained with 1 μg/mL 4,6 diamidino-2-phenylindole (DAPI) and mounted in Vectashield antifade solution (Vector Laboratories). Finally, slides were counterstained with DAPI and mounted in Vectashield. The slides were observed under an AxioScope A1 Imager fluorescent microscope (Carl Zeiss, Gottingen, Germany), and digital images were captured with an AxioCam MRc5 and analyzed using AxioVision v. 4.7 imaging software.

## Results

### Isolation and Characterization of Ty1-copia RT Sequences from *E. arundinaceus*

To characterize Ty1-copia RT sequences from *E. arundinaceus* genomic DNA, a pair of degenerate primers corresponding to the conserved Ty1-copia RT domain were used to amplify the Ty1-copia RT sequences. After recovery and cloning, a total of 94 independent clones were randomly selected for sequencing. Based on sequencing results, 70 sequences (74.5%) were found to have lengths ranging from 251 to 266 bp, and 24 sequences (25.5%) varied in length, ranging from 284 to 289 bp. Of the shorter Ty1-copia RT sequences, 65 out of 70 (92.9%) were 263 bp in length. All putative Ty1-copia RT sequences were translated into amino acid sequences, suggesting the presence of upstream TAFLHG and downstream YVDDM motifs, as well as a central conserved SLYGLKQ domain. BLAST comparisons of the obtained 70 Ty1-copia sequences with those in GenBank and EMBL databases revealed their sequence homology with known Ty1-copia RT retroelements from other plants. This indicated that Ty1-copia retrotransposons were distributed in *E. arundinaceus* genomes. In the longer Ty1-copia RT sequences, 15 out of 24 (62.5%) were 289 bp in length. However, despite possessing the upstream TAFLHG and downstream YVDDM motifs, these longer sequences did not share the central conserved domain of Ty1-copia RT sequences (SLYGLKQ), indicating they were not Ty1-copia RT sequences. Interestingly, all of these longer sequences were highly homologous (86%) to EaCIR1, a specific *E. arundinaceus*-derived subtelomere satellite sequence. Moreover, these sequences showed similarity (60%) with A19, a retrotransposon in *Oryza rufipogon*. This indicated that these sequences were not Ty1-copia RT sequences but possibly retrotransposon-like sequences. Hence, these non-Ty1-copia-related retrotransposons did not undergo phylogenetic analysis.

### Phylogenetic Analysis of Ty1-copia RT Sequences from *E. arundinaceus*

**FIGURE 1:**
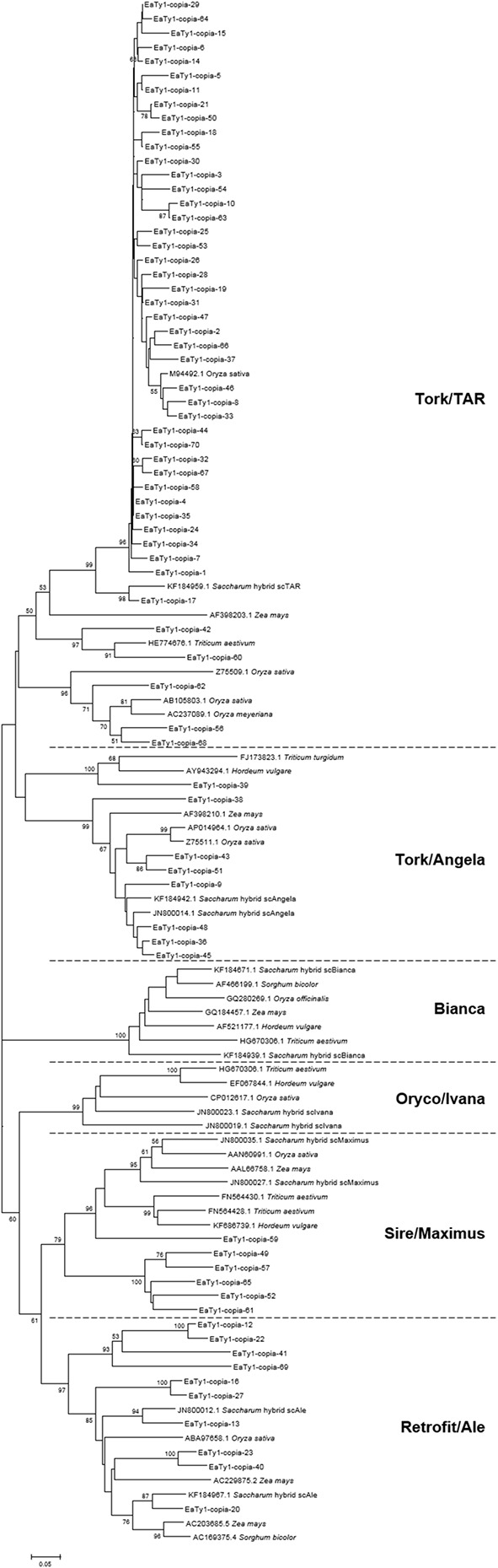
Neighbor-Joining tree of amino acid sequences based on alignment of Ty1-copia reverse transcriptase (RT) sequences from *Erianthus arundinaceus* with those from other graminaceous species (*Saccharum*, *Triticum*, *Hordeum*, *Oryza*, *Sorghum*, and *Zea*). Bootstrap values over 50 are indicated at the nodes.

To conform whether the ORFs of Ty1-copia sequences were defective or not, MACSE was used to detect disablements in ORFs. MACSE can detect nucleotide substitutions that result in stop codons and insertion or deletion of nucleotides that induce frameshifts (Ranwez et al., 2011). All identified 70 RT nucleotide sequences were manually inspected, and amino acid sequences encoded by the identified RT nucleotide sequences were deduced with consideration of spontaneous frameshift mutations. The amino acid sequences were further aligned to observe divergence, and the primer-binding regions were trimmed for phylogenetic analysis of Ty1-copia RT sequences from *E. arundinaceus* genome. To investigate the relationships among the isolated *E. arundinaceus* RTs with related Ty1-copia retrotransposons from other graminaceous species (*Saccharum*, *Triticum*, *Hordeum*, *Oryza*, *Sorghum*, and *Zea*), a Neighbor-Joining tree was constructed by aligning Ty1-copia RT amino acid sequences (**Figure 1**). A high level of sequence heterogeneity was observed among amino acid sequences of Ty1-copia RT from *E. arundinaceus*, and they were separated into four distinct evolutionary lineages (I–IV). Lineage I contained 46 *E. arundinaceus* Ty1-copia RT sequences with a highest average sequence similarity of 91%, which were clustered with scAngela, a Tork/TAR lineage of *Saccharum* (**Figure 1**). This might suggest that the Tork/TAR lineage makes up the largest proportions of Ty1-copia in *E. arundinaceus*. Among these Ty1-copia RT sequences, only 6 (13%) contained potentially functional Ty1-copia RT domains, whereas the remaining 40 (87%) possessed degenerate coding regions that contained frameshifts, stop codons, or deletions. The defective ORFs probably resulted in disruption of transpositional functionality of the element. The blastn analysis revealed Ty1-copia RT sequences of *E. arundinaceus* from lineage I had the highest similarity (88–90%) to other graminaceous species. Lineage II was comprised of eight *E. arundinaceus* Ty1-copia RT sequences with relatively high average sequence similarity (89%) clustered with scAngela, a Tork/Angela lineage of *Saccharum* (**Figure 1**); half of these sequences possessed stop codons or frameshifts. According to similarity search with blastn against the NCBI database, Ty1-copia RT sequences of *E. arundinaceus* from lineage II shared the relatively high sequence similarity (77–90%) to those of other graminaceous species. Lineage III consisted of six *E. arundinaceus* Ty1-copia RT sequences with an average sequence similarity of 78%, possessed disrupted ORFs, and were clustered with scMaximus, a Sire/Maximus lineage of *Saccharum* (**Figure 1**). A GenBank blastn search revealed Ty1-copia RT sequences of *E. arundinaceus* from lineage III was 70–74% identical to those from other graminaceous species. Lineage IV was homologous to scAle, a Retrofit/Ale lineage of *Saccharum* (**Figure 1**). This evolutionary lineage was composed of 10 *E. arundinaceus* Ty1-copia RT sequences with an average sequence similarity of 75%. All *E. arundinaceus* fragments from the Retrofit/Ale lineage, except for EaTy1-copia-12 and EaTy1-copia-20, represented potential pseudogenes (possessed stop codons or frameshifts). Ty1-copia RT sequences of *E. arundinaceus* from lineage IV were found to show significant similarity (72–80%) using blastn search. However, the Bianca and Oryco/Ivana lineages were not detected within the *E. arundinaceus* genome.

Given the available whole-genome sequence data in rice, sorghum, and maize, we compared the distribution and copy number of Ty1-copia RT sequences in *E. arundinaceus* with those in the three species. We found the majority of the aligned Ty1-copia RT sequences in *E. arundinaceus* existed in multiple copies and were dispersed on different chromosomes in the three species (**Table 1**). Additionally, there was clearly a tendency for the Ty1-copia RT sequences of the same lineage to be located on the same chromosomes in the same species (**Table 1**). However, the different distribution of Ty1-copia RT sequences from distinct lineages was observed in the three species (**Table 1**). For example, the Ty1-copia RT sequences from lineage I (Tork/TAR) were located on all chromosomes in rice, whereas the Ty1-copia RT sequences from this lineage were dispersed on some chromosomes in sorghum (chromosomes 2, 3, 4, 6, 9, and 10) and in maize (chromosomes 1, 2, 4, 5, 6, 9, and 10). Similarly, the Ty1-copia RT sequences from lineage II (Tork/Angela) were present on all chromosomes in rice and sorghum, but those from this lineage were found on chromosomes 2, 4, 7, 8, 9, and 10 in maize. Moreover, the Ty1-copia RT sequences from lineage III (Sire/Maximus) were widely distributed on all chromosomes in sorghum and most chromosomes except for chromosomes 9, 10, and 12 in rice, while those from lineage III (Sire/Maximus) were found on only chromosomes 1 and 2 in maize. Finally, the Ty1-copia RT sequences from lineage IV (Retrofit/Ale) were dispersed on some chromosomes in the three species (chromosomes 1, 2, 4, 7, and 12 in rice; chromosomes 1, 2, 3, 4, 6, 7, 8, and 9 in sorghum; chromosomes 2, 3, 6, 7, 9, and 10 in maize). The copy number of Ty1-copia RT sequences from distinct lineages varied greatly in the three species. For instance, most abundant in sorghum with 139 copies was the lineage II (Tork/Angela), while the similar copy number of this lineage was found in rice with 82 copies and maize with 91 copies, respectively. Similarly, both lineage III (Sire/Maximus) and lineage IV (Retrofit/Ale) in sorghum were present in 90 and 111 copies, respectively. In contrast, the copy number of these two lineages in rice and maize was much lower than that in sorghum. Lineage III (Sire/Maximus) was found in rice with 16 copies and maize with only 4 copies, as well as lineage IV (Retrofit/Ale) was present in rice with only 5 copies and maize with 40 copies. The Ty1-copia RT sequences from lineage I (Tork/TAR) had medium-copy number in rice with 67 copies and in maize with 59 copies, whereas this lineage was found in sorghum with 24 copies.

**Table 1:** Comparison of the distribution and copy number of Ty1-copia RT sequences in *Erianthus arundinaceus* with rice, sorghum, and maize.

Species	Lineage	Chromosome	Copy number
Rice	Tork/TAR	1, 2, 3, 4, 5, 6, 7, 8, 9, 10, 11, and 12	67
	Tork/Angela	1, 2, 3, 4, 5, 6, 7, 8, 9, 10, 11, and 12	82
	Sire/Maximus	1, 2, 3, 4, 5, 6, 7, 8, and 11	16
	Retrofit/Ale	1, 2, 4, 7, and 12	5
Sorghum	Tork/TAR	2, 3, 4, 6, 9, and 10	24
	Tork/Angela	1, 2, 3, 4, 5, 6, 7, 8, 9, and 10	139
	Sire/Maximus	1, 2, 3, 4, 5, 6, 7, 8, 9, and 10	90
	Retrofit/Ale	1, 2, 3, 4, 6, 7, 8, and 9	111
Maize	Tork/TAR	1, 2, 4, 5, 6, 9, and 10	59
	Tork/Angela	2, 4, 7, 8, 9, and 10	91
	Sire/Maximus	1 and 2	4
	Retrofit/Ale	2, 3, 6, 7, 9, and 10	40

### Relative Abundance in *E. arundinaceus* Genome and Chromosomal Distributions of Ty1-copia RT Sequences

**FIGURE 2:**
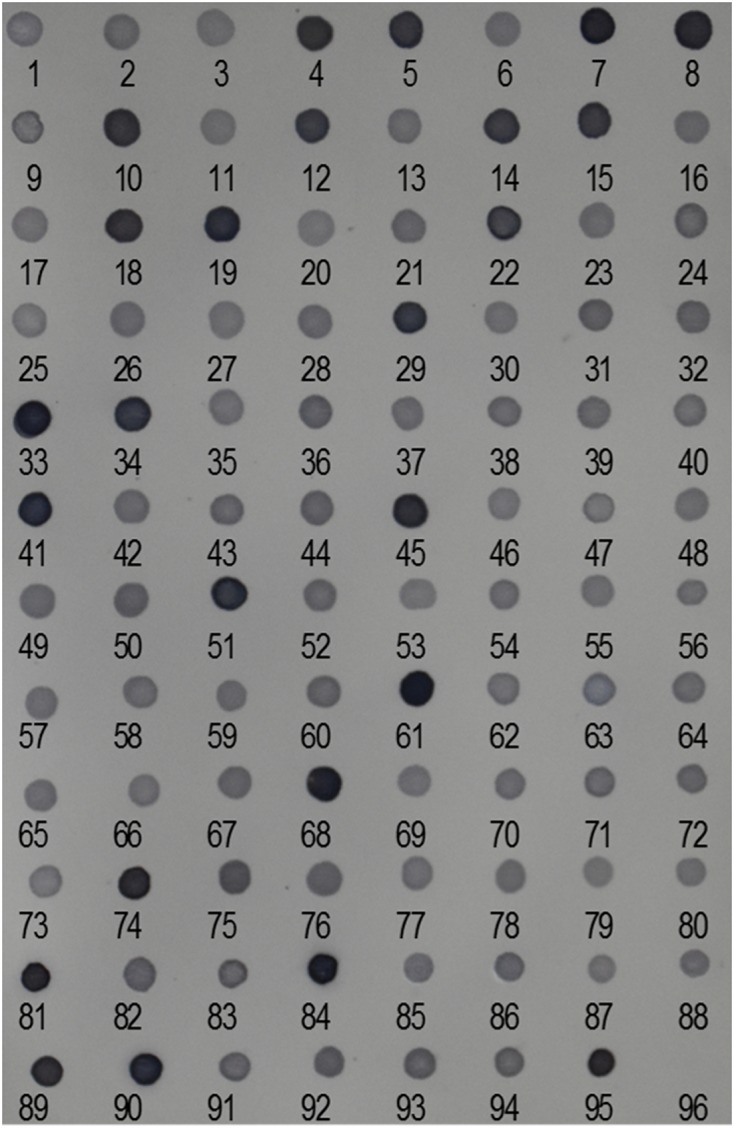
Relative abundance of Ty1-copia retrotransposons in the *E. arundinaceus* genome. Reverse dot-blot hybridization to Ty1-copia RT clones isolated from *E. arundinaceus* using genomic DNA of *E. arundinaceus* as a probe. Numbers indicate clone numbers. Nos. 95 and 96 are genomic DNA from *E. arundinaceus* as a positive control and double-distilled H_2_O as a negative control, respectively.

**FIGURE 3:**
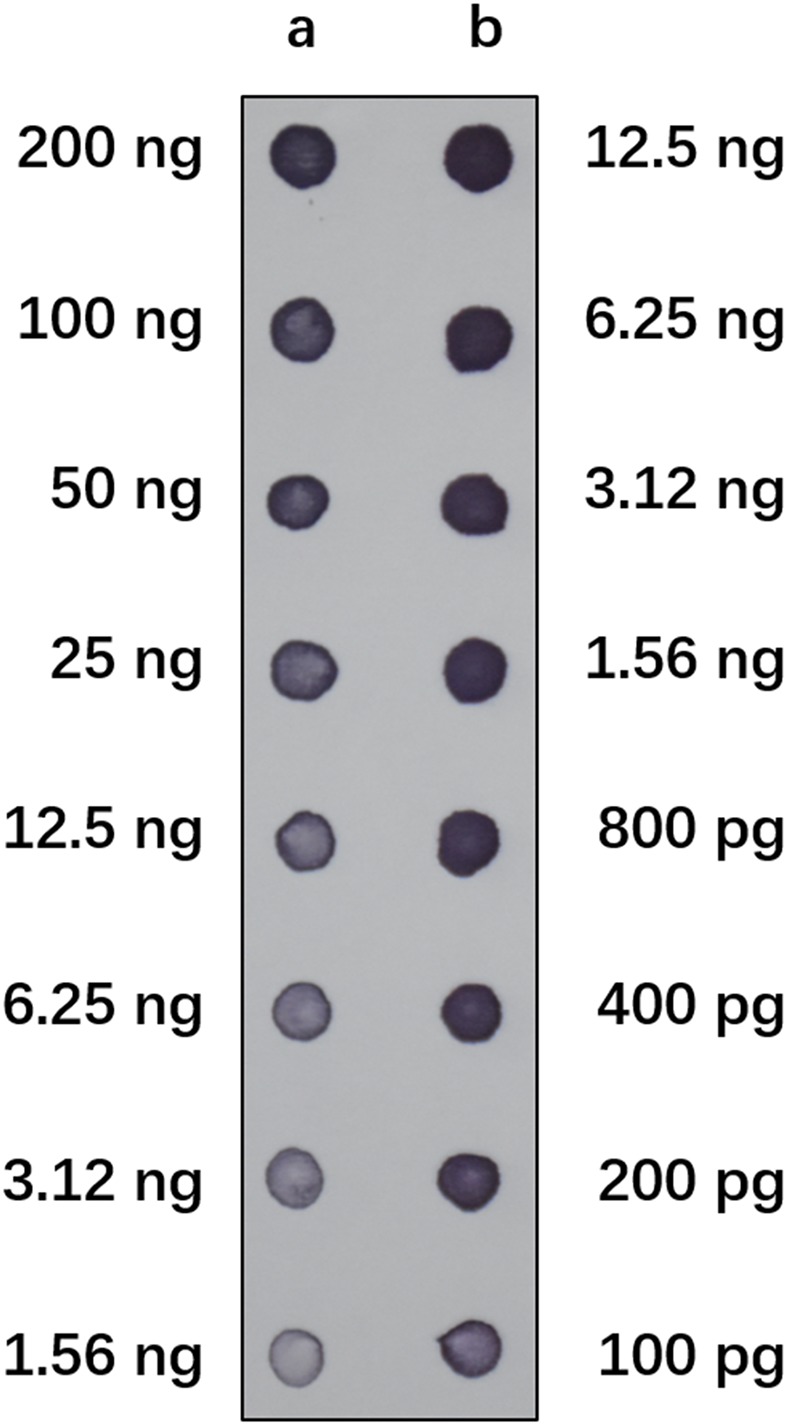
Estimation of the total copy number of Ty1-copia RT sequences in *E. arundinaceus* genome. Serial dilutions of genomic DNA from *E. arundinaceus* (row a) and plasmids containing clones of Ty1-copia RT sequences (row b) were blotted on a membrane. The filter was hybridized with the labeled polymerase chain reaction (PCR) probe containing clones of the Ty1-copia RT sequences.

We performed reverse dot-blot hybridization analysis to examine the relative abundance of isolated Ty1-copia RT clones from the *E. arundinaceus* genome. All subtelomere-associated non-Ty1-copia-related clones were found to have very strong hybridization signals (**Figure 2**), confirming that subtelomere-associated non-Ty1-copia-related retrotransposons were quite abundant in the *E. arundinaceus* genome. On the other hand, relatively weaker hybridization signals were obtained among the isolated Ty1-copia RT clones (**Figure 2**), indicating they had much lower copy number. To estimate the total copy number of these Ty1-copia retrotransposons in the *E. arundinaceus* genome, we performed a quantitative dot-blot assay using serial dilutions of the amplified clones as probes and genomic DNA from *E. arundinaceus*. The results indicated that their total copy number was approximately 4.5 × 10^3^ per haploid genome (**Figure 3**).

**FIGURE 4:**
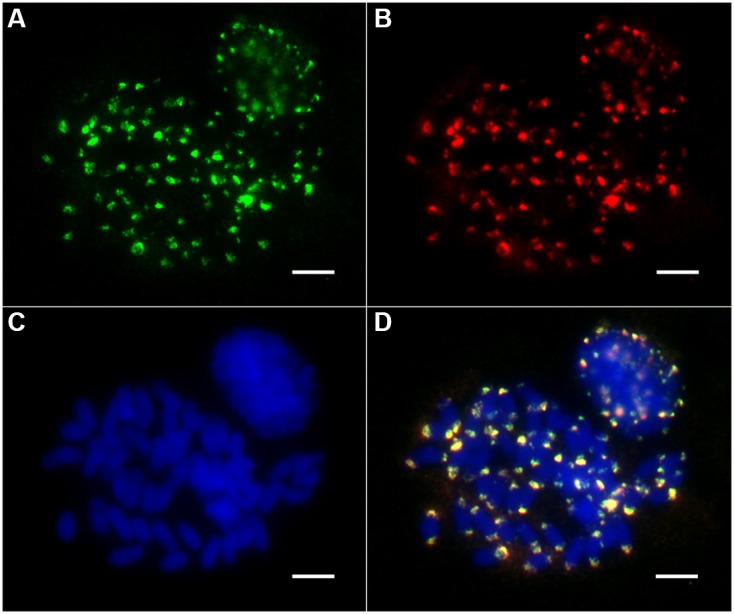
Chromosomal distribution of Ty1-copia retrotransposons from *E. arundinaceus* Hainan 92-77 (2*n* = 6*x* = 60). **(A)** The Ty1-copia RT sequences were labeled with DIG (green signal). **(B)** Non-Ty1-copia-related retrotransposons were labeled with biotin (red signal). **(C)** Chromosomes were counterstained light blue with 4,6 diamidino-2-phenylindole (DAPI). **(D)** Merged images of three signals. Scale bars = 5 μm.

To investigate the chromosomal distribution of both the non-Ty1-copia-related and Ty1-copia retrotransposons in the *E. arundinaceus* genome, we performed FISH on somatic metaphase chromosomes and interphase nuclei. The non-Ty1-copia-related retrotransposons were located exclusively in the distal regions at both ends of most somatic metaphase chromosomes, while FISH signals in interphase nuclei were concentrated in the heterochromatic regions (**Figure 4**). Intriguingly, we found that all Ty1-copia retrotransposons from the four lineages co-localized with the non-Ty1-copia-related retrotransposons in somatic metaphase chromosomes and interphase nuclei (**Figure 4**). These observations suggested Ty1-copia retrotransposons of the four lineages have similar distribution patterns in *E. arundinaceus* chromosomes.

## Discussion

As an important wild species of the *Saccharum* genus, *E. arundinaceus* has become one of the most popular germplasm sources for improvement of sugarcane. Nonetheless, little research has recently been focused on understanding the genetics and genomic evolution of *E. arundinaceus*. Retrotransposons make up a large fraction of genomes in higher plants, and these elements are thought to have contributed significantly to genome organization and evolution (McCarthy et al., 2002). The advent of PCR methods for amplifying conserved regions of retrotransposons has led to the proliferation of large number of molecular level studies of these elements on sequence evolution and phylogenetic relationships, especially of Ty1-copia retrotransposons from a wide range of plant taxa from monocotyledonous to dicotyledonous plants (Flavell et al., 1992; Pearce et al., 1996b; Goodwin and Poulter, 2002; Santini et al., 2002; Ma et al., 2008; Alipour et al., 2013; Kolano et al., 2013; Lee et al., 2013). Moreover, many investigations have focused on determining the chromosomal position of Ty1-copia retrotransposons and estimating their copy number (Pearce et al., 1996b; Alipour et al., 2013; Kolano et al., 2013; Lee et al., 2013).

In the current study, Ty1-copia retrotransposon sequences from the *E. arundinaceus* genome exhibited a high degree of heterogeneity in sequence and length. This is consistent with data reported for various other angiosperm species (Flavell et al., 1992; Pearce et al., 1996b; Ma et al., 2008; Alipour et al., 2013; Kolano et al., 2013; Lee et al., 2013). Moreover, the majority (>80 %) of Ty1-copia RT sequences are defective in *E. arundinaceus*. The defective ORFs probably resulted in disruption of transpositional functionality of the element, owing to the presence of stop codons and frameshifts. Although it remains unclear what causes the sequence heterogeneity, there are several possible explanations for it: (i) Due to lack of proof-reading ability, RT has a high error rate when transcribing RNA into DNA, and mutations continue to accumulate with each replication cycle when retrotransposons proliferate by a replicative mode of transposition (Gabriel et al., 1996; Keulen et al., 1997). (ii) The high heterogeneity of retrotransposons may be influenced by divergence during both horizontal transmission between distantly related species and vertical transmission down evolving lineages (Flavell, 1992). (iii) During periodic proliferation and burst of retrotransposons, the vast majority of retrotransposons are constantly truncated from the host genome through illegitimate and unequal homologous recombination (Devos et al., 2002; Tian et al., 2009). Defective elements may result in accumulation of mutations which create heterogeneous elements in the host genome. Unexpectedly, approximately a quarter of sequences belonging to the non-Ty1-copia-related retrotransposons were also identified from *E. arundinaceus* genome.

Although genomic sequence data from *E. arundinaceus* are not yet available, previous retrotransposon large-scale analyses in BAC sequences from sugarcane, a species closely related to *E. arundinaceus*, have provided an opportunity for better understanding of the diversification of Ty1-copia retrotransposons from the *E. arundinaceus* genome. To date, Ty1-copia families can be divided into six major common evolutionary lineages, namely Tork/TAR, Tork/Angela, Sire/Maximus, Retrofit/Ale, Oryco/Ivana, and Bianca (Wicker and Keller, 2007). In the current study, the 70 RT sequences of Ty1-copia families from *E. arundinaceus* were classified into four evolutionary lineages (Tork/TAR, Tork/Angela, Retrofit/Ale, and Sire/Maximus) out of six major ones (**Figure 1**). Of the four lineages identified, the Tork/TAR lineage contained the largest Ty1-copia retrotransposons within *E. arundinaceus*, followed by Retrofit/Ale, Tork/Angela, and Sire/Maximus. In fact, the number of Ty1-copia retrotransposons within each lineage may vary tremendously among various species. For example, Tork/TAR has the largest number of LTR retrotransposon families in soybean, but may be facing extinction in *Arabidopsis*, as only a single intact element of this lineage was found in the entire genome (Du et al., 2010). Sequence identity results have shown the Retrofit/Ale lineage has relatively low levels of nucleotide similarity (75%), whereas the Tork/TAR lineage showed high similarity (94%). Notably, we did not detect Ty1-copia retrotransposons from the Oryco/Ivana and Bianca lineages in *E. arundinaceus*. The absence of these two lineages in the *E. arundinaceus* genome may be explained by the relatively low representation in other angiosperms, judging from their low diversity where they have been detected from whole-genome sequencing and BAC library resources. For instance, the Oryco/Ivana and Bianca lineages were minor components of Ty1-copia retrotransposons in sugarcane (Domingues et al., 2012), which may explain its low representation in *E. arundinaceus*. The Bianca lineage was not found from whole-genome sequencing data even in soybean. Hence, certain Ty1-copia retrotransposon lineages may have been lost over evolutionary time. Moreover, another possible explanation is due to amplification bias of degenerate primers, resulting in inefficient amplification of targeted regions of the Ty1-copia RT sequences from other lineages. Fortunately, with the advent of next-generation sequencing from diverse graminaceous plants, numerous sequences of Ty1-copia retrotransposons from different lineages could be more widely available from whole-genome sequencing data or BAC sequences in graminaceous plants. Comparison of the Ty1-copia RT sequences from different lineages among diverse graminaceous plants has shown there are several conserved motifs among different lineages. In fact, comparison between the amino acid sequences of diverse graminaceous plants (*Saccharum*, *Triticum*, *Hordeum*, *Oryza*, *Sorghum*, and *Zea*) at these conserved motifs revealed a remarkable degree of conservation between these elements from the same lineages, but there were a few changes in amino acid sequences between these elements from different lineages (Supplementary Figure S1). To some extent, the divergence of these conserved motifs from diverse lineages could also explain why some lineages were not detected in *E. arundinaceus* when using these degenerate primers.

Due to the nature of the subtelomere-associated satellite sequences with high copy number, very strong hybridization signals were detected in non-Ty1-copia-related retrotransposons (**Figure 2**). In contrast, we found that all the Ty1-copia retrotransposons from *E. arundinaceus* exhibited similar abundance and relatively weaker signals (**Figure 2**). The copy number of the Ty1-copia retrotransposons from *E. arundinaceus* were nearly 4.5 × 10^3^ per genome (**Figure 3**), suggesting that retrotransposons might play a vital role in the genome evolution of *E. arundinaceus*. It is now well-established that retrotransposons are key drivers in the evolution of plant genome size (Devos et al., 2002; Tsukahara, 2009). Plant genomes either undergo downsizing by eliminating the transposed copies or tend to increase through bursts of retrotransposition. In different host genomes, the copy number of retrotransposons can vary considerably from several hundred elements to over one million. For instance, in plant species with relatively smaller genomes, such as *Arabidopsis* and rice, retrotransposons comprise less than 5% of the *Arabidopsis* genome (121 Mbp) and 17% of the rice genome [389 Mbp] (McCarthy et al., 2002; Pereira, 2004). Conversely, in plant species with medium and large genomes, retrotransposons constitute 75% of the maize genome (2300 Mbp) and 70% in the barley genome [5439 Mbp] (Vicient et al., 1999; Schnable et al., 2009). Even in the same host genome, the different lineages of Ty1-copia retrotransposons can vary enormously in copy number. A previous survey of Ty1-copia retrotransposons from soybean, *Arabidopsis*, and rice, all indicated that the Ale lineage has the tendency to evolve a wide variety of mostly very low-copy families, whereas the most ancient Bianca lineage is always found in multiple copies. It is well known that angiosperm polyploids possess a highly plastic genome structure, as manifested by tolerance to large-scale genomic changes, including retrotransposable element mobility. Although multiple copies of retrotransposons are defective in transpositional functions, there may be a very considerable number of transcriptionally active retrotransposons. *E. arundinaceus* is a wild species with various ploidy levels, including triploid, tetraploid, and hexaploid, which provide raw materials for researches of the role of retrotransposons on polyploidy genome evolution.

As expected, all the non-Ty1-copia-related retrotransposons were located in the subterminal heterochromatic regions at both ends of most metaphase chromosomes (**Figure 4**). Although non-Ty1-copia-related retrotransposons shared highly homology with the subtelomere-associated satellite sequence EaCIR1, the chromosomal distribution pattern of EaCIR1 was significantly different from that of the non-Ty1-copia-related retrotransposons. The hybridization sites of EaCIR1 sequences were distributed in subtelomeric regions at both ends of nearly half of the chromosomes, as well as in subtelomeric regions at one end of the other half of the chromosomes, while both ends of two chromosomes showed no hybridization signal (Alix et al., 1998). Unexpectedly, all the Ty1-copia retrotransposons from these four lineages were co-localized with the non-Ty1-copia-related retrotransposons (**Figure 4**). Notably, retrotransposon sequences have not been found in the nucleolus organizer regions in some plants (Kumar and Bennetzen, 1999). Therefore, we inferred that the Ty1-copia retrotransposons from the *E. arundinaceus* genome may only be located in the subterminal heterochromatic regions at both ends of chromosomes, excluding nucleolus organizer regions. In some cases, the Ty1-copia retrotransposons were dispersed throughout all of the chromosomes, clustering dominantly in the distal regions of the arms. The chromosomal distribution patterns of Ty1-copia retrotransposons have previously been described in some plants, including *Jatropha* and *Allium cepa* (Pearce et al., 1996b; Alipour et al., 2013). In addition, the chromosomal distributions of Ty1-copia retrotransposons exhibited distinct patterns in other species. For example, in *Beta vulgaris*, *Vicia faba*, and *Hordeum vulgare*, Ty1-copia retrotransposons were widespread and throughout the euchromatin, but less prevalent in heterochromatin regions (Schmidt et al., 1995; Pearce et al., 1996a; Suoniemi et al., 1996). Although sugarcane is a species closely related to *E. arundinaceus*, the Ale and Maximus lineages in sugarcane were found dispersed along chromosomes, whereas no FISH signals were detected for the Angela and Ivana lineages; the lack of signal was due to the low copy number of these two lineages (Domingues et al., 2012). This suggests evolutionary differences in the origin of heterochromatin between sugarcane and *E. arundinaceus* might influence the distribution of Ty1-copia retrotransposons. However, it is unknown why the distribution of Ty1-copia retrotransposons is different in various species. A likely explanation is that these Ty1-copia retrotransposons appear to a preferentially insert in more distal regions of chromosomes. Obviously, Ty1-copia retrotransposons were not distributed randomly along chromosomes in *E. arundinaceus*, and the distal regions were preferentially targeted by retroelement insertions, implying that these regions are possible hotspots for genomic changes in *E. arundinaceus*. Hence, Ty1-copia retrotransposons could be involved in heterochromatinization.

In the present study, we performed a concomitant survey of phylogenetic diversity, genomic abundance, and chromosomal distribution of Ty1-copia retrotransposons in the *E. arundinaceus* genome. The results presented here reveal the presence of a substantial amount of heterogeneous families of Ty1-copia retrotransposons, which are grouped into four lineages (Tork/TAR, Tork/Angela, Retrofit/Ale, and Maximus). These four different lineages of Ty1-copia families had similar abundance in the *E. arundinaceus* genome, and FISH patterns also confirmed that these retrotransposons were only located in the distal part of the ends of most chromosomes. Overall, our findings promote the understanding of the evolution of Ty1-copia retrotransposons in *E. arundinaceus*.

## Author Contributions

YH, JW, and ZD designed the research. YH, LL, and XH performed the experiments. YH, LL, XH, FY, YY, RC, and MZ analyzed the results. YH, ZD, and MZ wrote and revised the manuscript, and all authors read and approved the final manuscript.

## Conflict of Interest Statement

The authors declare that the research was conducted in the absence of any commercial or financial relationships that could be construed as a potential conflict of interest.

## Footnotes

1 http://www.ncbi.nlm.nih.gov/BLAST/

2 http://www.ebi.ac.uk/Tools/st/emboss_transeq/

3 https://phytozome.jgi.doe.gov/pz/portal.html#
