# Supplementary material for: Characterization, Genomic Organization, Abundance, and Chromosomal Distribution of Ty1-copia Retrotransposons in Erianthus arundinaceus
Source: Front Plant Sci. 2017 Jun 7;8:924. doi: 10.3389/fpls.2017.00924 (PMC5461294; doi:10.3389/fpls.2017.00924)
Supplement: Supplementary file 1 [file Image_1.pdf]

Supplementary Material

Characterization, Genomic Organization, Abundance, and Chromosomal Distribution of Ty1-copia Retrotransposons in *Erianthus arundinaceus*

Yongji Huang[1], Ling Luo[1], Xuguang Hu[1], Fan Yu[1], Yongqing Yang[1], Zuhu Deng[1, 4]*, Jiayun Wu[2,3]*, Rukai Chen[1], Muqing Zhang[4]

[1]National Engineering Research Center for Sugarcane, Fujian Agriculture and Forestry University, Fuzhou, China

[2]Guangdong Key Laboratory of Sugarcane Improvement and Biorefinery, Guangzhou, China

[3]Guangdong Provincial Bioengineering Institute, Guangzhou Sugarcane Industry Research Institute, Guangzhou, China

[4]Guangxi Collaborative Innovation Center of Sugar Industries, Guangxi University, Nanning, China

***Correspondence:**
Zuhu Deng
dengzuhu@163.com
Jiayun Wu
jiayunng@163.com

Supplementary Figure

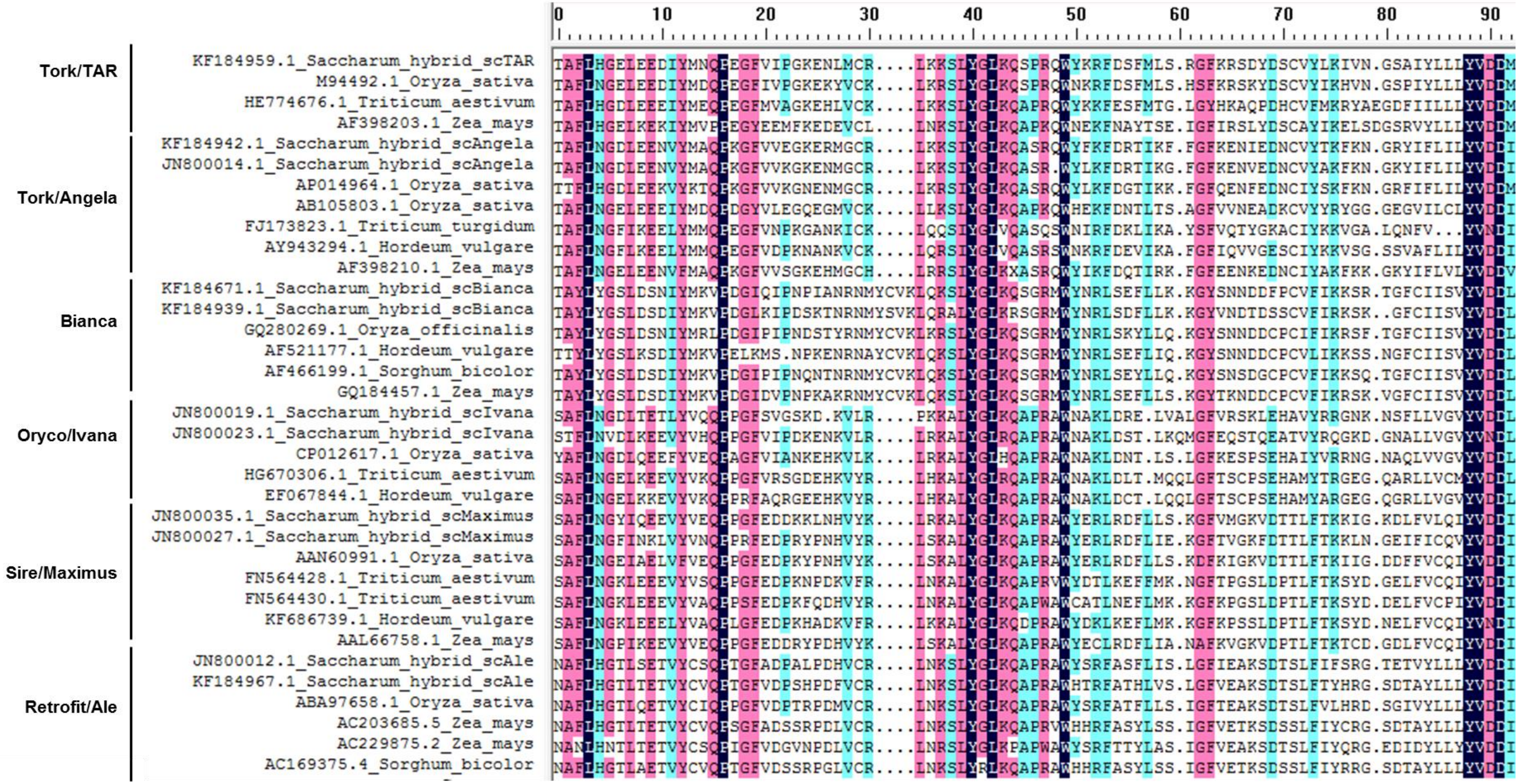

Supplementary Figure S1. Alignment of the amino acid sequences from graminaceous species (*Saccharum*, *Triticum*, *Hordeum*, *Oryza*, *Sorghum* and *Zea*) of Ty1-copia RT sequences.
